# Immunological Responses of Marine Bivalves to Contaminant Exposure: Contribution of the -Omics Approach

**DOI:** 10.3389/fimmu.2021.618726

**Published:** 2021-02-18

**Authors:** Teresa Balbi, Manon Auguste, Caterina Ciacci, Laura Canesi

**Affiliations:** ^1^Department of Earth, Environment and Life Sciences (DISTAV), University of Genoa, Genoa, Italy; ^2^Department of Biomolecular Sciences (DIBS), University of Urbino, Urbino, Italy

**Keywords:** bivalves, immunity, environmental stress, pollutants, biomarkers, -omics technologies

## Abstract

The increasing number of data studies on the biological impact of anthropogenic chemicals in the marine environment, together with the great development of invertebrate immunology, has identified marine bivalves as a key invertebrate group for studies on immunological responses to pollutant exposure. Available data on the effects of contaminants on bivalve immunity, evaluated with different functional and molecular endpoints, underline that individual functional parameters (cellular or humoral) and the expression of selected immune-related genes can distinctly react to different chemicals depending on the conditions of exposure. Therefore, the measurement of a suite of immune biomarkers in hemocytes and hemolymph is needed for the correct evaluation of the overall impact of contaminant exposure on the organism's immunocompetence. Recent advances in -omics technologies are revealing the complexity of the molecular players in the immune response of different bivalve species. Although different -omics represent extremely powerful tools in understanding the impact of pollutants on a key physiological function such as immune defense, the -omics approach has only been utilized in this area of investigation in the last few years. In this work, available information obtained from the application of -omics to evaluate the effects of pollutants on bivalve immunity is summarized. The data shows that the overall knowledge on this subject is still quite limited and that to understand the environmental relevance of any change in immune homeostasis induced by exposure to contaminants, a combination of both functional assays and cutting-edge technology (transcriptomics, proteomics, and metabolomics) is required. In addition, the utilization of metagenomics may explain how the complex interplay between the immune system of bivalves and its associated bacterial communities can be modulated by pollutants, and how this may in turn affect homeostatic processes of the host, host–pathogen interactions, and the increased susceptibility to disease. Integrating different approaches will contribute to knowledge on the mechanism responsible for immune dysfunction induced by pollutants in ecologically and economically relevant bivalve species and further explain their sensitivity to multiple stressors, thus resulting in health or disease.

## Introduction

### Bivalves, Pollutants, and Immunity

Bivalves, the second largest group in Mollusca, show a large diversity in anatomical structure and size, physiology and behavior, and adaptation to different environments. Moreover, several bivalve species represent an important food source for humans and are of considerable economic value in aquaculture in estuarine and coastal areas (1). Coastal environments are not only characterized by natural fluctuations in different environmental parameters but are also generally subjected to a variety of anthropogenic pressures, including the release of pollutants from different sources. Due to their worldwide distribution, sedentary nature, and filter-feeding habits, knowledge on the biochemical and physiological adaptations of bivalves, and their feasibility for experimental handling, have been long recognized as “sentinel” organisms for the evaluation of responses to chemical contamination of the aquatic environments (2–4). These responses, called biomarkers, have been widely and thoroughly investigated in species representative of bivalve groups that are of main commercial interest (mussels, oysters, and clams) and have been applied worldwide in marine biomonitoring programs to identify early signs of exposure to contaminants and to quantify the health status of coastal waters (2–4). Biomarkers are also currently applied to evaluate responses of marine bivalves to emerging classes of pollutants, such as microplastics (5), nanomaterials (6), and pharmaceuticals (7). Among the biomarkers, particular attention is given to those that can reveal processes whose disturbances from the molecular, cellular, tissue, and whole organism level can be reflected at a higher level of biological organization (population and community level). These include physiological functions, such as energy metabolism, reproduction, and immune response [(8) and references quoted therein].

The bivalve's affords efficient protection against different potential invaders, based on the activity of circulating immunocytes, the hemocytes, which can recognize potential pathogens and foreign particles through phagocytosis and different cytotoxic reactions, secreting soluble factors in the hemolymph fluid (8–14). Bivalve hemocytes, thanks to their capacity to cross epithelial barriers and the characteristics of the open circulatory system, play pleiotropic roles in other functions, such as nutrient transport, biomineralization, and reproduction (15–17). As more information is available on the molecular and functional traits of hemocyte function (18, 19), understanding the potential impact of pollutant exposure on bivalve immune defense is gaining increasing attention.

### Immune Response of Bivalves to Contaminant Exposure

The first overview of the immune responses of marine invertebrates, including bivalves, to environmental perturbation, including contaminant exposure, was provided by Ellis et al. (20). In this work, available information was also discussed within the context of “ecological immunology,” which refers to the role of environmental stressors on the evolution of the immune response (21). Ellis et al. (20) analyzed available data on cellular/humoral immune responses to environmental stress evaluated across different species, obtained utilizing immune biomarkers as classical parameters: changes in hemocyte abundance and subtypes, morphology or viability, and functional parameters, from phagocytic activity as a proxy for immunocompetence, to oxyradical and nitric oxide production, release of antimicrobial enzymes, etc., evaluated by standard biochemical and cellular techniques. The importance of evaluating expression of individual antimicrobial peptides (AMPs) and immune-related genes by quantitative PCR was also underlined, but the application of gene expression to bivalve immunity was not yet straightforward enough (20). This study underlined the following: (1) differences among species in response to a single stressor, intraspecific responses to different stressors, or to single stressors at different levels of exposure; (2) the importance to evaluate immune responses of a wider range of species from different phyla; (3) the need to investigate a greater number of environmental stressors, alone and in combination; (4) the influence of seasonal factors in determining the immune response to environmental stress; and (5) the need to evaluate how changes in immune responses induced by environmental stress will impact host–pathogen interactions. These points are still crucial in understanding the potential impact of environmental contaminants on immune defenses of marine invertebrates.

The first review article focusing on pollutants and immunity in marine bivalves was from Renault (22), where different classes of inorganic and organic pollutants, including emerging contaminants such as pharmaceuticals and nanoparticles (NPs), were considered. Also, in that case, most of the available data were obtained evaluating classical immune biomarkers as described above; in particular, this study underlined how research on potential immunotoxicity of NPs was also focused on the identification of their mechanisms of action in bivalve hemocytes (22). Actually, studies on NPs and bivalve immunity are of interest since they also represent a unique, particulate form of pollutants that can be utilized as a model of non-self material to investigate the response of the innate immune system (6, 23).

In the last few years, the number of publications on the effects of pollutants on bivalve immunity has greatly increased, and no updated review papers are available yet. However, a systematic review of the advances in this field has become an extremely difficult task, not only because of the large amount of datasets obtained with a number of chemicals (tested in a variety of experimental settings and different bivalve species), but also the number and type of immune biomarkers measured, and the type of responses observed to be increasing the concentration of pollutants. With regard to this latter point, an example was first provided by data obtained in the model bivalve, the mussel *Mytilus galloprovincialis*, on immune responses evaluated after exposure (*in vitro* and *in vivo*) to a number of estrogenic compounds, alone and in mixture (24). Classical functional immune parameters (phagocytosis and lysozyme release) showed no changes, stimulation/inhibition, at increasing concentrations of chemicals. Subsequent data obtained in *M. galloprovincialis* with other classes of contaminants (such as heavy metals, nano-oxides, and nanoplastics) supported these observations (24–27). In this respect, functional immune biomarkers behave as “bell-shaped” biomarkers, that is, those showing increases and subsequent decreases at increasing stress conditions, such as the many general and specific biomarker responses utilized in biomonitoring (28). Thanks to the progress in the identification of immune-related genes and protein sequences in different bivalve species, more recent data on functional responses are reported together with expression of individual genes in hemocytes. Examples of stimulation/inhibition of different functional parameters and up- and down-regulation of selected immune-related genes have been described in different bivalves exposed to a variety of contaminants (23, 29–34), thus reflecting the capacity of different species to adequately respond to a certain amount of stress.

Overall, available data generally show that whatever individual parameter is measured (cellular or humoral/functional or molecular), each can change in response to contaminants depending on the conditions of exposure (concentration, time of exposure, and experimental setting). In this light, in analogy with the general biomarker approach, where integrated multiple biomarker responses are utilized to identify the response to environmental stress (35, 36), only the determination of a battery of different functional and molecular immune biomarkers will be able to describe the overall impact of contaminants on bivalve immunocompetence.

Moreover, this scenario is further complicated by the general capacity of the bivalve immune system to cope with changing environmental conditions. The *in vivo* effects of contaminants can often be reversible, suggesting the establishment of compensatory mechanisms (37). Recent data indicate that mussels can establish mechanisms of tolerance in their immune defenses after repeated exposure to microplastics and nanoplastics (23, 38). Finally, interactive effects of pollutants with natural abiotic variables (temperature, salinity, and pH) on immune responses have been described in different bivalves (39–44). In the natural environment, the immunomodulatory properties of a variety of pollutants at low and variable concentrations, in the presence of changing environmental factors, may be crucial in affecting the health status of bivalves, contributing to mass mortalities in the most susceptible species. Renault (22) pointed to the need of investigating in detail the potential interference of pollutants in host–pathogen interactions, in an attempt to understand the complex relationship between contaminant exposure, immunocompetence, and susceptibility to disease. To reach this goal, a great experimental effort is needed, utilizing different experimental settings: *in vitro* exposure of hemocytes to screen the immunomodulatory properties of different contaminants and to identify the main mechanisms of action at the cellular level; *in vivo* exposure of individuals at realistic concentrations for each contaminant, trying to reach conditions compatible with environmental exposure; finally, and most importantly, to evaluate the response of pollutant-exposed hemocytes or individuals to challenge with different pathogens, to gain an insight on the subsequent effects on host–pathogen interactions.

In this context, *in vivo* experiments, in addition to the obvious experimental relevance, offer a further advantage to understand how immune homeostasis is affected by pollutant exposure. These studies allow not only the measurements of immune biomarkers in hemocytes and hemolymph serum but also to investigate the immune response at the tissue level. This is important when considering the capacity of bivalves to accumulate different classes of contaminants in different tissues (i.e., the gills and the digestive gland or hepatopancreas). In particular, the gills, which through their filter-feeding activity represent the first contact with water-borne pathogens, are also the first site of uptake and accumulation of water-soluble pollutants (i.e., heavy metals). In this light, the possibility to evaluate the immune response also at the tissue level, not only as hemocytic infiltration as a sign of inflammation (45), but also by measuring tissue-specific molecular immune responses to contaminant accumulation, will greatly contribute to the understanding of how bivalves are able to orchestrate the immune response at the whole organism level. To this end, high-throughput molecular techniques represent a powerful tool that is not fully exploited yet. Recent contributions of -omics approaches to bivalve immunity (18, 46–49) offer an extraordinary potential to identify critical molecular targets and pathways of contaminants and, in general, the biochemical response of organisms to environmental stress.

## Impact of Pollutants on Bivalve Immunity in the Postgenomic ERA

Due to the enormous development of DNA high-throughput sequencing, in the last decade draft genomes have been generated for different molluscan species, including several bivalves (mussels, oysters, and clams) (50). Although producing quality assemblies for molluscan genomes has proven extremely challenging, due to several factors (i.e., genome size, composition of repetitive elements, and levels of heterozygosity), the increasing amount of data has increased the quality of the assemblies and annotations [reviewed in Gomes-dos-Santos et al. (50)]. Studies on the genome of selected bivalve species will contribute to unraveling the molecular mechanisms underlying multiple adaptations to different environments (49) and will greatly improve the interpretation of transcriptome datasets that, in comparison, are relatively easy to generate.

In the last decade, the application of -omics approaches in ecotoxicology has been proposed and applied in order to identify toxicity pathways, to more precisely quantify classical biomarkers, to identify novel ones, and to draw adverse outcome pathways (AOPs) (51–53). In parallel, several studies have been focused on the application of -omics, mainly transcriptomics, to bivalve immunity (46). In comparison, publications on immune proteomics and metabolomics are currently limited, but their expansion should be facilitated as more complete genome sequences and transcriptome datasets become available for different species.

However, the -omics approach has only been utilized in the last few years to investigate the effects of contaminants on bivalve immunity. Although a PubMed search for “Bivalve Immunity & Pollutants” gave 29 hits for “transcriptomics,” 14 for “proteomics,” and seven for “metabolomics,” respectively, only a few of these publications focused on immune responses. In this mini-review, we made a first attempt to highlight the main advancements in knowledge, the limitations of available data, and future developments of the application of the -omics approaches to evaluate the impact of contaminants on bivalve immunity in health and disease.

### Transcriptomics

As mentioned above, the transcriptomic approach has been largely applied to the study of disease processes in marine bivalves [reviewed in Nguyen and Alfaro (48)]. Although a detailed overview of a large number of available data is outside the scope of this work, these studies greatly contributed to the increasing knowledge on innate immunity, bivalve–pathogen interactions, and mechanism of resistance to disease and pathogen virulence.

Transcriptomics has also been increasingly utilized in evaluating the effects of exposure of several inorganic and organic contaminants in different bivalve species. A number of studies identified a significant impact of a wide range of contaminants (heavy metals, pesticides, pharmaceuticals, and hydrocarbons), on different pathways, such as response to stress, redox balance, metabolism, biotransformation of xenobiotics, apoptotic processes, and immunity (41, 54–61). However, these data were obtained from the analysis of the transcriptome at the tissue level (digestive gland, gills, and gonads), indicating that changes in immune transcriptome reflect a general perturbation of homeostatic processes. Although this information represents an enormous increase in knowledge on changes in immune gene expression induced by pollutants, which is crucial in understanding the most susceptible pathways, only a few publications were focused on immune responses. Moreover, transcriptomic data are seldom associated with the determination of functional immune parameters and do not explain how the physiological response of the organism to infection may subsequently be affected.

In *Mytilus edulis*, the effects of cadmium (Cd) were evaluated in an *in vitro* model of mussel hemocytes by transcriptomics (RNA-seq and DGE analysis) and the determination of functional parameters, namely phagocytosis and hemocyte viability (62). The results showed among the Cd-regulated genes, several toll-like receptors, and genes involved in phagocytosis and apoptosis pathways, underlying a link between molecular and functional responses.

Recently, the molecular responses of hemocytes to both cadmium exposure and pathogen stimulation were investigated *in vivo* in the oyster *Crassostrea gigas* (63). Transcriptome data revealed specific molecular responses to cadmium exposure in the absence or the presence of *Vibrio splendidus*. The results showed the activation of a neuro-endocrine-immune regulatory network in response to Cd^2+^ and vibrio exposure; in particular, bacteria stimulation could reverse or strengthen the expression pattern of some Cd-responsive genes (63). Although no functional immune parameters were evaluated, at present, this is the only transcriptomic study reporting data on the effects of contaminant exposure on the response to pathogen challenges in bivalves. Overall, although transcriptomics represents the most utilized -omics tool in the field of immunity and in response to contaminants in bivalves so far, further studies are needed to establish a link between contaminant exposure, transcriptional changes in hemocytes, and alterations in immune responses toward potentially pathogenic microorganisms.

### Proteomics

Examples of the application of proteomic studies on bivalve immunity are available in different species (59, 64–68). These studies not only add information on the protein repertoire expressed by hemocytes and hemolymph plasma, including proteins potentially involved in recognition, binding and intracellular and extracellular destruction of microorganisms, but also can contribute to identifying potential new immune biomarkers that can be used for the evaluation of the impact of environmental pollutants on immune processes.

Environmental proteomics focus on the detection of changes in the level of individual proteins/peptides induced by environmental stressors. A good example of their application to immune responses to natural stressors was provided by a study on the oyster *C. gigas* exposed to ocean acidification (OA) and challenge with *V. splendidus*, alone and in combination (69). The results showed that several hemocyte functional parameters [cell number and viability, phagocytic activity, and production of reactive oxygen species (ROS)] were affected by OA and that *V. splendidus* infection exacerbated the impaired immune responses under OA exposure. Proteomic analysis revealed differential responses to OA stress and pathogen challenge, alone or in combination. OA appeared to act *via* a generalized stress response by causing oxidative stress, subsequently leading to cell injury through disruption of the cytoskeleton, protein turnover, energy metabolism, and immune responses. In contrast, *V. splendidus* alone induced similar cell injuries in the absence of oxidative stress and could act directly on the immune system. Combined exposure to OA and vibrio challenge presented a similar, but stronger effect on the proteome compared with OA treatment alone, indicating synergistic effects on oyster immune functions, with OA exposure potentially increasing the risk of *V. splendidus* infection (65). The application of a similar approach would represent a significant advancement in the study of the impact of contaminants on immune responses.

The application of environmental proteomics in different bivalve species, as sentinel organisms in biomonitoring pollution of the aquatic environment, was first extensively reviewed by Campos et al. (70). After describing the main techniques utilized, the Authors summarized available knowledge on the identification of biochemical markers of exposure to a number of pollutants (i.e., heavy metals, hydrocarbons, phthalates, polybromodiphenyl ethers, and bisphenol A). The potential for identification of subproteomics, redox proteomics (protein modification due to oxidative stress), or posttranslational protein modification, was also underlined. A recent work reviewed available proteomic data on aquatic organisms, including the mussel *Mytilus* as the most studied bivalve species, in response to pollutants; the results indicate that both heavy metals and organic contaminants generally induce alterations of structural and metabolic proteins, antioxidant enzymes, and immune-related proteins (71). Advantages and drawbacks of aquatic pollution biomonitoring utilizing the proteomic approach were discussed, underlying as the main future development the need to focus on alternative protein isoforms, protein interactions, and posttranslational modifications, to increase knowledge on the relationship between proteins and their functions, as well on the regulation of protein signaling networks (71). However, it is only recently that proteomic data has revealed the specific impact of pollutants on the immune response. Green et al. (72) investigated the effects of microplastics (MPs) on *M. edulis* in a long-term outdoor mesocosm experiment; the exposure to different types of MPs resulted in changes in the humoral components of mussel hemolymph, including many proteins involved in detoxification, metabolism, and structural development. Among immune-related proteins, members of the complement C1q domain-containing proteins were affected by certain types of MPs. Although no immune or general stress biomarkers were evaluated, MPs significantly reduced the production of byssal threads and their strength of attachment. The authors hypothesized that these immunological changes may be due to physical abrasion from ingested MPs (72).

In the Sydney rock oyster *Saccostrea glomerata*, the effects of a neonicotinoid insecticide broadly utilized in agriculture, imidacloprid (IMI), on immune responses were investigated under two salinity regimes, evaluating both functional immune parameters and changes in proteome (73). The IMI exposure significantly altered the expression of several hemolymph proteins, including an increase in extracellular superoxide dismutase (SOD), severin, ATP synthase β subunit, and different stress response proteins (heat shock proteins, serine/threonine-protein kinase DCLK3, and peroxiredoxin-1). The results indicated that the oyster immune system was affected by IMI at environmentally relevant concentrations (73).

Overall, the application of proteomic studies focused on hemocytes and plasma, together with measurements of different functional hemolymph parameters, in contaminant-exposed bivalve species, may reveal specific responses induced by pollutant exposure, thus contributing to identifying those protein components involved in host-pathogen interactions that are more susceptible to the action of chemical contaminants.

### Metabolomics

During the last decade, environmental metabolomics has been applied in ecotoxicological studies to investigate the mechanisms of action of single contaminants and mixtures (74–78). Metabolomics allows for the identification of low molecular weight (LMW) metabolites (50–1500 Da) whose production and levels can vary with the physiological and pathological state of cells, tissues, organs, or whole organisms, as well as with development [(79) and references therein]. Metabolomics can offer an excellent snapshot of what is actually happening in the organism at a given time (80) and could be considered as a tool for early identification of ongoing homeostatic changes. Data obtained are highly accurate and reproducible, sample preparation requires minimal effort and time, and acquisition of spectral data is quite fast (81). However, spectra assignment is still one of the main challenges of metabolomics (74, 82). Moreover, in several organisms, including bivalves, due to the current lack of knowledge on their metabolism, few metabolites can be annotated or identified (79). Finally, metabolomics responses can be hindered by both environmental variables (i.e., temperature, pH, predation, food availability, etc.) and endogenous factors (i.e., gender, age, size, genetic, etc.) (79).

Metabolomics represents an excellent tool to investigate the effects of contaminants in aquatic organisms, especially if integrated with classical biological assays and/or other -omics techniques. However, to date, only one study was focused on hemocyte responses. In the clam *R. philippinarum*, cadmium, exposure induced alterations in total hemocyte counts and oxyradical production in a dose-dependent manner (83). Metabolomic analysis in the hepatopancreas showed that Cd^2+^ exposure induced immune stress and disturbance in energy metabolism.

Moreover, most metabolomic data obtained at the tissue or whole organism level indicate an alteration of immune-related processes in bivalves in response to contaminants. The effects of 17α-ethinylestradiol (EE_2_) on the unionid mussel *Lampsilis fasciola* were evaluated by traditional behavioral and reproductive endpoints and a metabolomic approach (84). Interestingly, based on the traditional reproductive endpoints, EE_2_ exposure induced fewer adverse effects than hypothesized. In contrast, metabolomic studies showed that EE_2_ significantly affected several metabolic processes in gills, leading to a reduction in energy reserves. Moreover, a modification of a set of metabolites involved in the immune response, among others, was observed. In the clam *R. philippinarum*, arsenite (As) and arsenate induced oxidative stress in the digestive gland. A metabolomic analysis demonstrated that both chemical forms of As induced immune stress, as shown by the increase in branched-chain amino acids (valine, leucine, and isoleucine) and osmotic stress (85). Cong et al. (86) showed that ammonia exposure of the clam *R. philippinarum* could cause not only lysosomal destabilization, but also metabolic disorders, malformations of gill structures, and changes in neurotransmitters, thus resulting in an alteration in feeding, respiration, and immune function. A metabolomic approach highlighted the disruption of metabolic pathways in the mussel *M. galloprovincialis* exposed to a wastewater treatment plant (WWTP) effluent (79). A common response observed in both males and females was represented by the main change in glycerophospholipid levels, possibly caused by oxidative stress, which could lead to reproductive disorders; in addition, gender-specific metabolic alterations in some polar lipids and kynurenine pathway were observed, suggesting a disturbance in the energy metabolism and immune system only in males. An integrated metabolomic and proteomic approach was utilized to evaluate the effects of Cd and As(V) on the early larval stages of *M. galloprovincialis* (87). Metabolomic data responses indicated an alteration in energy metabolism and osmotic regulation in response to both Cd and As. Proteomic analysis showed that Cd exposure induced several alterations at the protein level, thus leading to immune and oxidative stress, disturbance in nucleic acid metabolism, and cellular injury. A similar approach was utilized to study the effects of benzo(a)pyrene (BaP) on the gills of the pearl oyster *Pinctada martensii* (88). The results indicated that the two -omics techniques were complementary to demonstrate that the toxic effects of BaP were mainly due to alteration of immune response, osmotic regulation, and energy metabolism. Overall, available data underline the potential contribution of metabolomics in the identification of the immune function as a potential target of contaminant exposure in bivalves.

### Lipidomics

With the rapid advances in mass spectrometric detection, significantly increasing resolution and accuracy, untargeted lipidomic analysis represents a powerful tool for the identification of all detectable lipids and their metabolites, including unknown chemicals (89). In a recent study, a comparative analysis of lipid profiles was performed in four edible bivalve species (including clams, oysters, and mussels), which led to the identification of more than 600 different lipids belonging to 14 classes (90). Although this study was essentially carried out to establish a relationship between the nutritional value of each species and their lipid composition, the application of lipidomics will provide the baseline data for further studies on the effects of pollutants on the lipidome of other species utilized in biomonitoring programs. To date, the only available data are those obtained from oysters (*Crassostrea hongkongensis*) exposed to copper (Cu) (91). The observed changes in the lipidome profile in the digestive gland of Cu-exposed oysters were analyzed in aspects of membrane dynamics, lipid signaling, and energy metabolism; among the changes in different lipid classes, copper exposure induced phospholipid remodeling, and increased in polyunsaturated fatty acids that represent precursors of inflammatory mediators (91). Although the correlation between an imbalance in lipid homeostasis and the abundance of different molecular species and host defense is a poorly explored area of research (92), the possibility that pollutant exposure may affect immunomodulatory lipids in bivalves represents an intriguing field of investigation. However, the effects of contaminant exposure on hemocyte lipidomics related to immune functions remain unexplored to date.

### Metagenomics

High-throughput sequencing technologies also provide the ability to fully characterize the diversity of complex microbial populations (i.e., the microbiome) in environmental matrices, both at the taxonomic (e.g., DNA barcoding, 16S or 18S rDNA sequencing) and functional (e.g., metagenomics, metatranscriptomics, metabolite production, and gene pathway analysis) levels (46). It is now recognized that the innate immune system plays an important role in shaping the composition of the microbial communities associated with the host into configurations that can be not only well-tolerated but also beneficial for its metabolism; in turn, the microbiota integrate into the physiology of the host and, through its interactions with the innate immune system, can influence multiple homeostatic processes (93). In this light, it has been proposed that this issue should be considered in the broader context of the impact of anthropogenic activities, including chemical pollution, on different ecosystems (94).

The rich and complex microbiome of bivalves has been shown to be influenced by host genetics, environmental conditions, stress, and infection (14, 52, 95–100). An alteration of bivalve microbiota, in both tissues and hemolymph by environmental changes and/or stressful conditions, has been associated with changes in health status and higher susceptibility to disease (58, 59, 99, 101–107). A recent study showed that in oysters, the composition of bacterial communities in the hemolymph is more strongly shaped by environmental factors than by the genetic background of the host (108).

However, few there is not a lot of data available that focuses on the effects of pollutant exposure on the microbiome of bivalve hemolymph and on the possible relationship with the immune defenses of the host. We have recently shown that in *M. galloprovincialis, in vivo* exposure to nanoparticles induced significant shifts in the composition of microbial communities of the hemolymph, at the same time inducing changes in immune parameters, with distinct effects observed with different NPs (30, 109). Titanium dioxide NPs (nTiO_2_) induced an overall stimulation of immune responses, which was associated with an almost complete disappearance of *Vibrio* and *Psychrobium* from mussel hemolymph. These data indicate that nTiO_2_-induced activation of immune defenses may contribute to creating an unfriendly medium for the most sensitive bacterial communities present in hemolymph (30). On the other hand, polystyrene nanoplastics negatively affected different immune parameters, at the same time increasing the relative abundance of *Arcobacter*-like, *Psychrobium*, and *Vibrio*, suggesting that the downregulation of immune defenses may favor potentially pathogenic bacteria (109).

To decipher the factors underlying susceptibility to diseases and mass mortality in the field, Milan et al. (110, 111) proposed a broader approach on gene expression profiling and functional analyses of microbial communities based on the combination of RNA-sequencing and 16S microbiota analyses. In the Manila clam, *R. philippinarum*, tissue microbiota were affected by the interactions between seasonal variables and exposure to pollutants (110). Moreover, the results obtained in the striped venus clam, *Chamelea gallina*, indicate that opportunistic pathogens may take advantage of compromised host immune pathways and defense mechanisms by chemical exposure, thus contributing to periodic mortality events (111).

These studies will further our understanding of how exposure to different types of contaminants may shape the interactions between the host immune defenses and the associated microbiota in bivalves, thus affecting the health status of key species in coastal ecosystems that are subjected to natural environmental fluctuations and the impact of human activities.

## Conclusions and Perspectives

The major advances, current perspectives, and future directions of the application of the three main -omics approaches (transcriptomics, proteomics, and metabolomics) in bivalve hemocytes and hemolymph aquaculture research have been recently reviewed (48). The authors underlined how data obtained on the characterization of these multilevel responses to pathogenic infections can provide valuable information on the mechanisms that drive the innate immune system in response to stress, and on the complex host–pathogen–environment interactions across bivalve species. In this light, the development of epigenomic studies in marine invertebrates are also promising in evaluating the role of epigenetic modifications (DNA methylation, histone modifications, chromatin remodeling, and non-coding RNAs) on immune responses, and how these processes may be affected by environmental stress, thus affecting bivalve susceptibility to disease (112).

However, when we look at the state of the art on the application of -omics technologies to evaluate the effects of pollutants on immune responses of bivalves, only a few studies demonstrate a clear relationship between contaminant exposure and alterations of immune responses (Table 1). This underlines the need for specific studies focused on contaminant-induced alterations of immune function.

**Table 1:** Summary of available publications focused on the application of -omics to evaluate the effects of pollutants on bivalve immune responses.

	**Contaminant**	**Exposure**	**Species**	**Endpoints**	**References**
Transcriptomics	Cadmium	*In vitro* 21 h Cd 10^−9^**–**10^−3^ M	*Mytilus edulis*	Hemocyte RNA-seq, phagocytosis, viability	(62)
	Cadmium, cadmium/*Vibrio splendidus*	1 d, 100 μg/L CdCl_2_, bacteria 10^6^ CFU/mL	*Crassostrea gigas*	Hemocyte RNA-seq	(63)
Proteomics	Microplastics	Outdoors mesocosm 52 d, 25 μg/L	*Mytilus edulis*	Hemolymph proteins	(72)
	Imidacloprid	*In vivo*, 4 d (0.01, 0.1, 1 μg/L)	*Saccostrea glomerata*	Hemocyte functional parameters, hemolymph proteins	(73)
Metabolomics	Cadmium	*In vivo* 2 d, 20 and 200 μg/L	*Ruditapes philippinarum*	Hemocyte functional parameters, digestive gland metabolomics	(83)
Metagenomics	n-TiO_2_	*In vivo* exposure, 4 d 100 μg/L	*Mytilus galloprovincialis*	Hemocyte functional parameters, hemolymph microbiome	(30)
	Nanoplastics amino-modified nanopolystyrene	*In vivo* exposure, 4 d, 10 μg/L	*Mytilus galloprovincialis*	Hemocyte functional parameters, hemolymph microbiome	(109)

**Figure 1:**
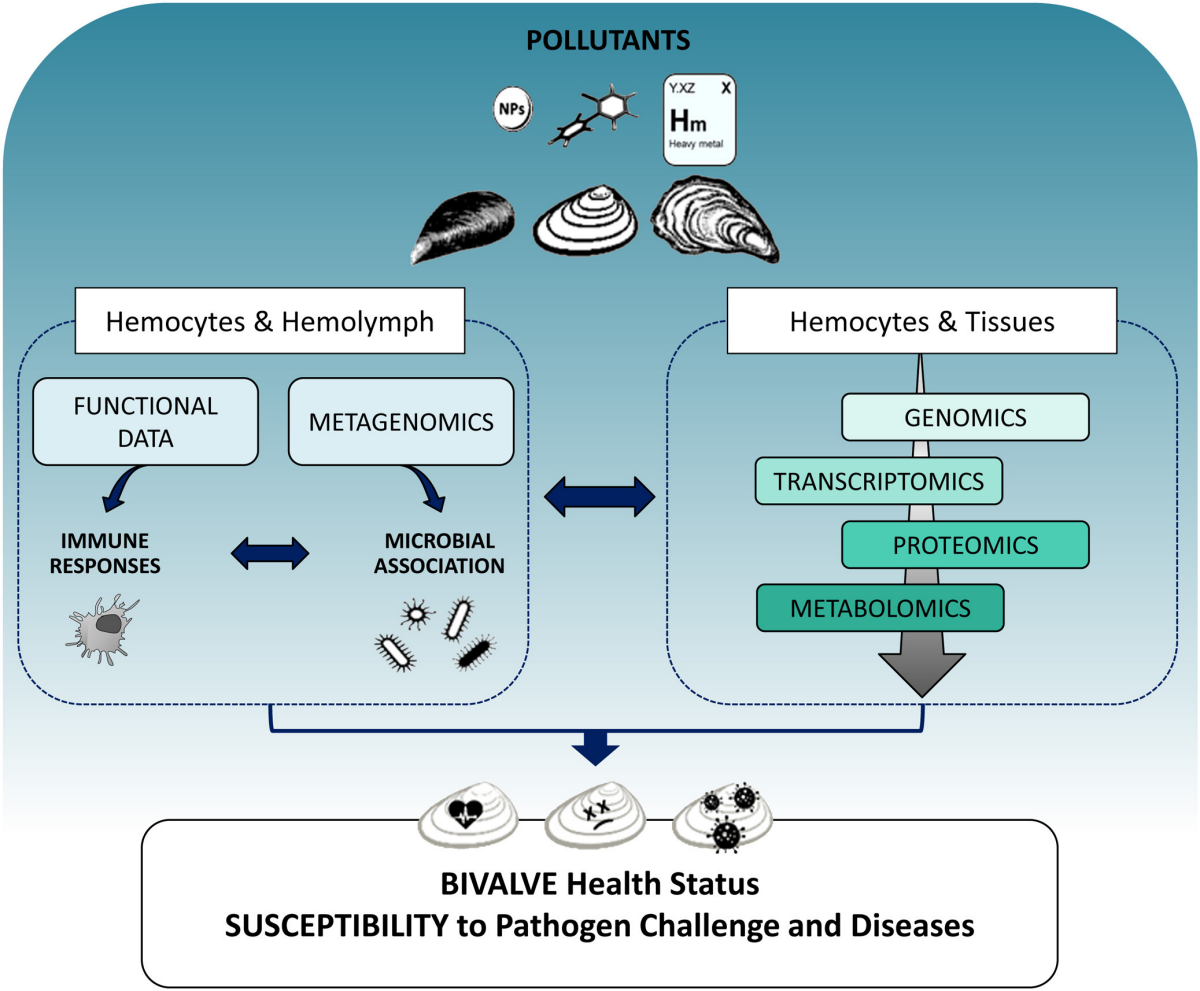
Schematic overview showing the integration of different -omics approaches in evaluating the impact of pollutants on the immune responses of bivalves and their consequences on organism health. Left: The effects of different contaminants (heavy metals, organic xenobiotics, and nanoparticles) can be evaluated on the main components of the immune system, hemocytes, and hemolymph plasma, determining functional immune responses as classical immune biomarkers. The application of metagenomics to hemolymph samples can provide an estimation of how contaminant exposure may shape the interactions between innate immune defenses and the associated microbial communities. Right: Different -omics tools approaches applied to both hemocytes and tissues can reveal perturbations of immune responses as changes in gene and protein expression networks and metabolic profiles related to immune processes at cellular and tissue level and can identify immune targets for different classes of pollutants. The overall information will help understanding how contaminant exposure can affect the capacity of different bivalves to cope with pathogen challenge, thus increasing their susceptibility to disease, with possible consequences on the health status of natural populations and aquacultured species.

Although the integration of different -omics approaches would greatly expand our knowledge on the bivalve immune system, and its responses to contaminant exposure, the need for complex methodologies and the analysis of large-scale datasets from different -omics platforms, currently makes this approach unfeasible in environmental monitoring. However, the differently expressed molecules that have been identified through -omics studies can be used as candidate immune biomarkers with applications in biomonitoring of environmental pollutants as well as in aquaculture for early disease diagnosis. At present, integrating data on functional immune responses and susceptibility to pathogen challenge with the application of individual -omics platforms at cellular and tissue level appears to be the most suitable approach to better define the mechanisms underlying the immune response to pollutants and their significance for the health status of bivalves (Figure 1). Understanding the capacity of bivalves to coordinate their immune defenses in response to contaminants will contribute to issues related to public health, food safety, and aquaculture.

## Author Contributions

LC conceived and designed the review. CC collected and analyzed available data. MA and TB designed the artwork. LC, MA, and TB critically wrote the manuscript. All authors contributed to manuscript revision, read, and approved the submitted version.

## Conflict of Interest

The authors declare that the research was conducted in the absence of any commercial or financial relationships that could be construed as a potential conflict of interest.
